# An integrative approach to discern the seed dispersal role of frugivorous guilds in a Mediterranean semiarid priority habitat

**DOI:** 10.7717/peerj.7609

**Published:** 2019-10-11

**Authors:** Diana Carolina Acosta-Rojas, María Victoria Jiménez-Franco, Víctor Manuel Zapata-Pérez, Pilar De la Rúa, Vicente Martínez-López

**Affiliations:** 1 Department of Zoology and Physical Anthropology, Faculty of Veterinary, University of Murcia, Murcia, Spain; 2 Senckenberg Biodiversity and Climate Research Centre (BiK-F), Frankfurt am Main, Germany; 3 Ecology Area, Department of Applied Biology, Faculty of Experimental Sciences, University Miguel Hernández, Elche, Spain; 4 Department of Ecological Modeling, UFZ–Helmholtz Centre for Environmental Research, Leipzig, Germany; 5 Department of Ecology and Hydrology, Faculty of Biology, University of Murcia, Murcia, Spain

**Keywords:** DNA barcoding, Mutualistic networks, Vertebrate frugivorous guilds, Complementary seed dispersal, Priority habitat 5220*, Mediterranean

## Abstract

Seed dispersal is an essential process to maintain the viability of plant populations, and understanding this ecological process allows management strategies to be developed to conserve ecosystems. European Union priority habitat 5220* is defined as “Mediterranean arborescent shrubland with *Ziziphus lotus*” and it represents a favorable microclimate within the severe climatic conditions typical of the semiarid south-eastern region of the Iberian Peninsula. Therefore, the study of seed dispersal in this priority habitat by different frugivorous guilds, is a challenge for its conservation. In this study, we have characterized a mutualistic network of seed dispersal that is mediated by vertebrates (mammals and birds) in the protected habitat 5220*. The aims of this study were to: (i) identify the seed disperser community; (ii) analyze the relative role of key species in the dispersal process; and (iii) compare the functional ecology of the seed dispersal process between mammals and birds. As such, we collected animal faeces to determine seed dispersers taxonomy, identifying the mammals through the visual aspect of the faeces and the birds by DNA barcoding. In the case of birds, we also collected regurgitated seeds in which the disperser species was also identified through molecular techniques. This allowed us to build-up a mutualistic network and to identify the relative role of these animals in seed dispersal. Our results showed that mammals and birds fulfilled complementary roles in seed dispersal, with birds representing the main dispersers of key plants within the 5220* habitat, and mammals the main dispersers of human-cultivated plants. Herein, we provide a useful approach with relevant information that can be used to propose management policies that focus on restoring the threatened 5220* habitat, promoting the role of birds to disperse key species that structure plant communities of this priority habitat.

## Introduction

Seed dispersal is an essential ecological process in the viability of plant populations, since it enables the mobilization of seeds from the parental plants toward sites where they can germinate and establish (Howe & Smallwood, 1982). In Mediterranean shrublands and in some tropical dry forests, between 50% and 70% of plant species rely on animals to disperse their seeds (Jordano, 2013). Today, the vulnerability of many ecosystems has been enhanced because of anthropic activities and natural phenomena (Cardinale et al., 2012; Newbold et al., 2015, 2016), as well as due to environmental effects like climate change (Ruxton & Schaefer, 2012). These disturbances often disrupt the functioning of ecosystems, including processes such as pollination and seed dispersal, which could provoke the local extinction of some species (Traveset, González-Varo & Valido, 2012). Preserving ecological processes like seed dispersal is important for the conservation of habitats, not least because it can palliate the effects of fragmentation by connecting natural remnants (Emer et al., 2018), and it may help plants to escape from stress factors such as global warming (González-Varo, López-Bao & Guitián, 2017a; Naoe et al., 2016). Therefore, studying the complex plant-animal interactions that may drive seed dispersal is crucial to assess ecosystem services and their integrity (Harvey et al., 2017).

The study of mutualistic seed dispersal networks allows plant-animal interactions to be explored and it provides a framework through which conservation strategies can be designed (Costa et al., 2016). Such approaches help identify the animal species that disperse the seeds of different plant species (Bascompte & Jordano, 2007; Jordano, 1987), and hence, evaluating the role of each taxa in seed dispersal providing an indication of the specific contribution of animals and plants to ecosystem maintenance. Mammals and birds are the two main taxa responsible for dispersing the seeds of plants with fleshy-fruits (Bello et al., 2017) and thus, most studies have focused on these frugivorous guilds to study seed dispersal (e.g., mammals: Peredo et al., 2013; Stevenson et al., 2002; and birds: Saavedra et al., 2014; Spotswood, Meyer & Bartolome, 2012). Although a number of studies have focused on the contribution of frugivores (both mammals and birds) to the dispersal of the seeds of certain fleshy-fruited plant species (Escribano-Ávila et al., 2012; Jordano et al., 2007; Li et al., 2016; Martínez, García & Obeso, 2008), there are few studies centered on seed dispersal networks at the community level, i.e.: involving all plants with fleshy-fruits and more than one animal class (but see Muñoz et al., 2017; Timóteo et al., 2017).

One reason for this taxonomic bias in mutualistic network studies is related to the different techniques to infer frugivory interactions, these involving animals with a wide variety of behavioral and morphological traits (Martínez, García & Obeso, 2008). For example, birds are usually sampled by methods that require individual capture with mist-nets and the recovery of seeds by dissection of their faeces (Martínez-López et al., 2017), or through focal observation of fleshy-fruited plants to register the removal of fruits by different species (Albrecht et al., 2014). However, these studies require long sampling periods and they may be biased by the specific characteristics of the dispersal vectors (e.g., different rates of detection: Kéry et al., 2010). For instance, the elusive behavior of mammals means they are hardly ever seen in the field (Gittleman et al., 2001), although their frugivory habits can be inferred by studying the content of their faeces, as different species can be identified by the shape and the size of such traces (Putman, 1984). More recently, molecular techniques have been applied to study bird seed dispersal, using seeds from faeces as templates to amplify target sequences (González-Varo, Arroyo & Jordano, 2014). Consequently, the fact that frugivorous mammals and birds can be taxonomically determined using non-invasive techniques makes it more feasible to build ecological networks that will provide additional information on ecosystem functioning.

According to the European Directive 92/43/EEC, habitat 5220* (hereafter, priority habitat 5220*) is defined as a “Mediterranean arborescent shrubland with *Ziziphus lotus* (L.) Lam.” and it is a protected priority habitat (Tirado, 2009) based on the presence of “productivity islands” in landscapes where food resources could be scarce. Indeed, these islands offer a favorable microclimate within the severe climatic conditions typical of the semiarid Southeast region of the Iberian Peninsula (Zapata et al., 2014). However, this semiarid shrubland is often present in landscapes with a high anthropogenic pressure due to urban development and agricultural holdings (García et al., 2007; Tirado, 2009). Moreover, the elevated temperature and scarce precipitation in these semiarid and arid ecosystems slow down any ecological succession (Buisson, Corcket & Dutoit, 2015; Rey Benayas et al., 2015).

Despite its recognized importance, only 49% of these semiarid shrublands unique to the Iberian Southeast are contained in the Natura 2000 Network (Márquez-Barraso et al., 2015). Moreover, their conservation becomes more pressing if we consider that more than 90% of the protected surface of this habitat is within only one of the “Sites of Community Importance” (SCIs: SCI—Cabo de Gata-Níjar, Almería, Spain). In fact, a loss of more than 40% of the potential habitat of one of the most representative species *Maytenus senegalensis* (Lam.) Exell, has been detected in this habitat 5220* (Mendoza-Fernández et al., 2015). As such, understanding how seed dispersal can mitigate extreme fragmentation will enable more effective conservation strategies to be planned, better guaranteeing persistence. However, as far as we know, this issue has only been studied in this priority habitat in relation to the interaction between the red fox *Vulpes vulpes* L. and *Z. lotus* (Cancio et al., 2016, 2017), highlighting the fundamental role of the fox as seed disperser of *Z. lotus* in this semiarid ecosystem. Indeed, they found that fragmentation caused a decline in *Z. lotus* fruits consumption by foxes which triggered a collapse in its seed dispersal.

In this study, we wanted to characterize the ecological interactions involved in the dispersal of seeds from fleshy-fruit species of the priority habitat 5220*, a semiarid shrubland within the SCI—Sierra de la Fausilla, located in the Southeast of Spain (Murcia). We specifically aimed to: (i) identify the seed disperser community of habitat 5220*; (ii) analyze the relative role of each frugivorous species (i.e., species which consume fruits independently of the feeding habits) in the seed dispersal process; and (iii) determine whether different frugivore taxa (mammals and birds) are complementary or redundant in terms of the seed dispersal associated with a priority habitat earmarked for biodiversity conservation. In order to address these results, we applied an integrative approach which combines classical field sampling (vegetation sampling, and species determination by visual analysis of faeces) with molecular techniques (DNA barcoding), as well as seed dispersal networks. We hypothesized that different frugivorous guilds are involved in the seed dispersal in the priority habitat. Moreover, we suspect that different vertebrate disperser taxa fulfil dissimilar roles in the dispersal of fleshy-fruit shrub seeds in the habitat 5220*. As such, we hope to disentangle the context-dependent complexity of this priority Mediterranean habitat which would enable the design of adequate management policies for its conservation and restoration.

## Materials and Methods

### Study area

**Figure 1:**
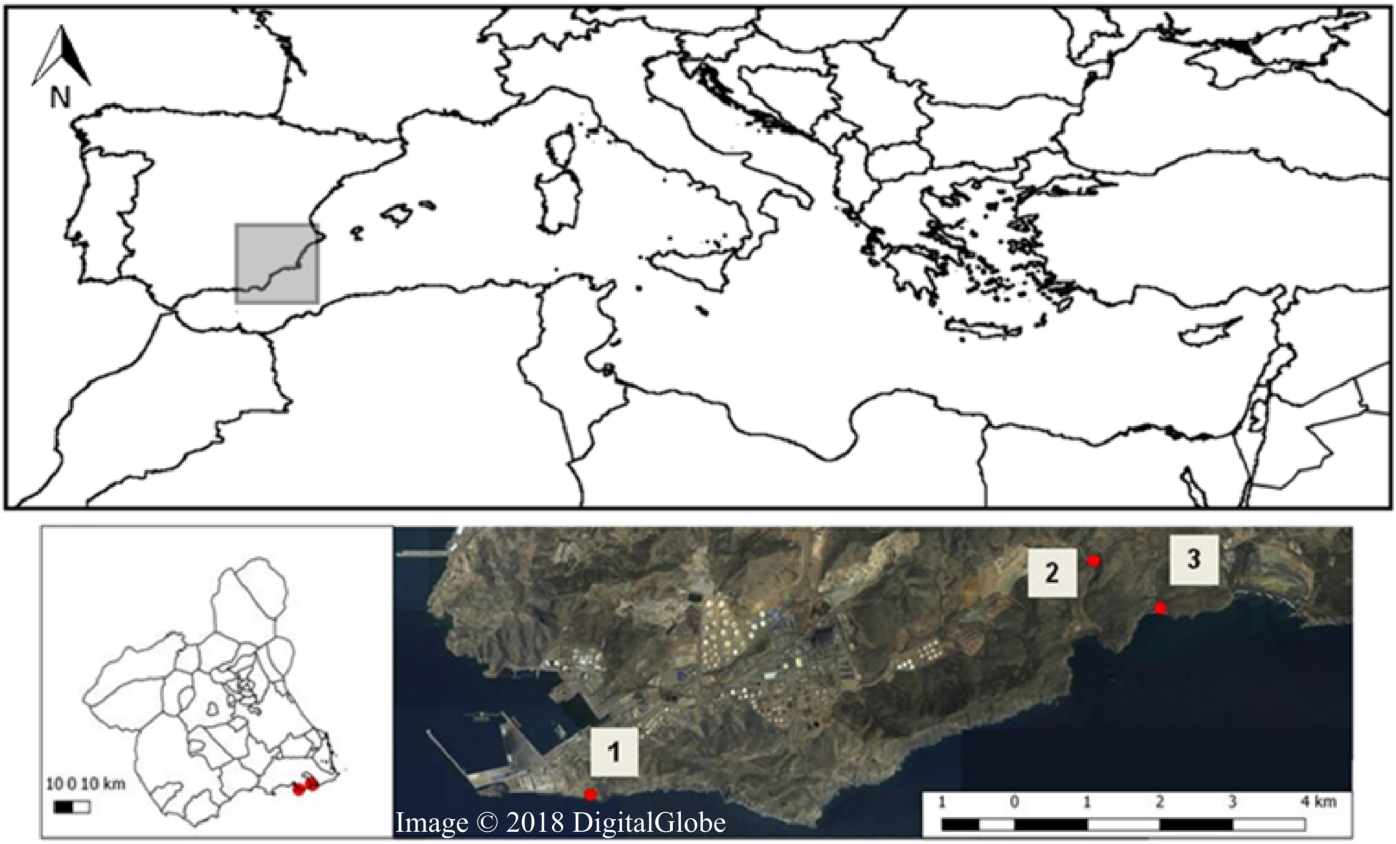
Map of Sierra de la Fausilla (Murcia Region, SE Spain) showing the three sampling sites: (1) Escombreras, (2) Gorguel, and (3) Cola de Caballo (Map from Map data © 2018 Google, Digital Globe).

The study was carried out in the SCI—Sierra de la Fausilla (Southeast of the Iberian Peninsula, Fig. 1), a coastal mountainous system of cliff morphology in the south-eastern region of the province of Murcia. This protected area located in the Mediterranean biogeographic region, belongs to the biogeographical province referred to as Murcia Almeriense, in which the most arid landscape in the Iberian Peninsula can be found (García et al., 2007). Covering 865.26 ha, the Sierra de la Fausilla was declared a SCI in the Natura 2000 Network (ES6200025: April, 1999) and a Special Protection Area (SPA ES000199: March 152 2000). Four types of priority habitat of community interest can be found in this protected area: 5220* (Mediterranean arborescent shrubland with *Z. lotus*), 1510* (Mediterranean salt steppes), 6110* (Rupicolous calcareous or basophilic grasslands of the Alysso-Sedion albi) and 6120* (Xeric sand calcareous grasslands) (Comunidad Autónoma de la Región de Murcia, 2005). In this study, we focused specifically on the semiarid shrubland habitat 5220*, which refers to pre-desert Mediterranean shrubland that grows from sea level to 300 m (Tirado, 2009) (Fig. S1), with limestone soils in depressions, riverbeds and zones of subsurface currents. The plant communities in this habitat are characteristically spiny species with tiny leaves that grow in the dry season, shaping islands of biodiversity that offer suitable microenvironments for the germination of other plant species (i.e., a nursery, Mendoza-Fernández et al., 2015), and shelter and food for animals. A large number of these Ibero-African plant species (i.e., species distributed in the North of Africa and in the South of the Iberian Peninsula) are fleshy-fruited shrubs of tropical or subtropical origin, considered relics of the wetter past climatic conditions (e.g. *Z. lotus*, *Periploca angustifolia* Labill., *Lycium intricatum* Boiss., *Maytenus senegalensis*, *Withania frutescens* (L.) Pauquy. and *Pistacia lentiscus* L.) (Sánchez-Gómez & Guerra, 2003). Their fruits are key trophic resources for frugivorous vertebrate animals as they provide both nutrients and hydration (Robbins, 1983).

In this sampling area, we selected three sites situated more than one km apart from each other in a region stretching from the coast Southeast of Cartagena to Cabo de Palos and bordering the Valley of Escombreras to the north: Zone 1: Escombreras; Zone 2: Gorguel and Zone 3: Cola de Caballo (Fig. 1). From the second half of the last century, this valley has undergone drastic physiognomic changes that have led to its current occupation by a large petrochemical refinery complex and some local industries. Moreover, there are non-endemic plant species associated to gardens in anthropogenic areas and crop plant species from oldfieds in the area.

### Plant community composition and sampling of frugivore-dispersed seeds

We sampled the local plant community to determine which plant species could potentially be dispersed by animals at the three study sites. Vegetation sampling was carried out on circular plots of 100 m^2^ (nine plots at each sampling site, 27 in total), in which all fleshy-fruited woody plant species were counted to determine their frequency of occurrence following the protocol developed previously (Zapata & Robledano, 2014).

The three sampling sites were visited fortnightly from September to December 2016 (six field trips) to collect animal-dispersed seeds (defecated in the case on mammals and defecated or regurgitated in the case of birds). Sampling procedures were performed during the peak abundance for fleshy-fruited plants in Mediterranean shrublands (Jordano, 1988). We looked for animal faeces (mammals and birds) and regurgitated seeds from birds in five fixed 100 m transects at each sampling site that were distributed in order to cover the landscape heterogeneity of the study areas. One transect in each study area included a dirt road, since many mammalian species have a preference for defecating in such anthropogenic structures (Suárez-Esteban, Delibes & Fedriani, 2013). We made a preliminary classification of the mammalian and bird faeces in the field, then each faecal sample was photographed next to an object reference for posterior identification. All samples were labeled with the date and site and stored in an airtight bag. Mammalian faeces were examined under a laminar flow chamber on plastic trays in the laboratory. These seeds were separated, washed with distilled water, dried at room temperature, and then stored at 5 °C for taxonomic classification. In the case of bird-dispersed seeds, we collected the faecal and regurgitated samples in sterile tubes and stored them at −20 °C for identification through molecular techniques. The plants dispersed by both animal classes were identified by the seed’s external morphology, comparing this with a reference collection of seeds of fleshy-fruited shrubs.

### Determination of seed dispersers

The taxonomic classification of mammal species dispersing seeds in Sierra de la Fausilla was achieved by analyzing photographs and the characteristics of the faeces collected, and by comparing our material with information available in trail and footprint guides (Rubines, 2015). These classifications were confirmed by a researcher with experience in the collection and processing of mammalian faecal samples. Seeds in bird faeces were separated from faecal material to be used in DNA extraction in this way reducing PCR inhibitors and increasing PCR yield (see Marrero et al., 2009 for rationale). Dispersers from bird-dispersed seeds were identified by DNA barcoding achieved by amplifying the animal DNA that remained on the seed surface after passage through the gut or regurgitation (González-Varo, Arroyo & Jordano, 2014). This molecular technique is based on amplification of a fragment of mitochondrial DNA (*cox1*: cytochrome c oxidase subunit I, Hebert, Ratnasingham & De Waard, 2003), which allows closely related species to be distinguished. Genomic DNA was extracted from seeds with the NucleoSpin Tissue extraction Kit (Macherey-Nagel, Düren, Germany). For PCR reactions, COI-fsdF and COI-fsdR primers were used following the PCR profile described previously (González-Varo, Arroyo & Jordano, 2014). PCR reactions were performed in a total volume of 12.5 µl, containing 2.5 µl of PCR buffer (5×), 0.5 µl of each primer (0.04 µM), 0.312 µl of BSA (0.5 mg/ml bovine serum albumin; Roche Diagnostics, Barcelona, Spain), 0.2 µl of Taq polymerase (1 U/Tube; Bioline, London, UK), and 6.48 µl of ultrapure water. As the study sites were close to the sea, implying high humidity, the DNA of many samples was degraded and hence, we used specifically designed primers for such cases: COI-fsd-degF and COI-fsd-degR (González-Varo et al., 2017b). Sequence edition and alignment was performed with Mega7 (Kumar, Stecher & Tamura, 2016), and “BOLD: The Barcode Life of Data System” (Ratnasingham & Hebert, 2007) was used to identify the bird species to which each sequence belonged.

### Data analysis

The density of fleshy-fruited species and the seed rain were analyzed in the three sampling sites using a non-parametric Kruskal–Wallis test. To determine whether the plant communities dispersed by mammals and birds were significantly different to those established in the sites studied, we assessed the data of the species dispersed by each taxon in relation to the density of the plants species found in vegetation samples, performing a Permutational Multivariate Analysis of Variance (PERMANOVA) with a Bonferroni correction, and using Bray–Curtis distances and 9,999 permutations. We graphically represent the three plant communities—plant species recorded in vegetation samples and plant species dispersed by birds and mammals—with non-metric multidimensional scaling (NMDS) (Anderson, 2014). Data of species dispersed by each taxon were considered as the number of interactions between each plant and each animal species, that is, the number of times that viable seeds of a plant species were found in animal-dispersed samples. Moreover, we calculated both *Chao1* and *Chao2* estimators in order to appraise the diversity of plant species that was observed for the sampling plots of native plants, and identified in seed dispersal of birds and mammals (Sarmah, 2017). *Chao1* estimates the total diversity in a sample of sites using abundance data, whereas *Chao2* estimates species richness based in presence and absence information (Escribano-Avila, 2019). We considered each sampling plot of native plants and the mammal and bird dispersed samples as the sampling effort units for the samplings of native plants and plant species dispersed by both animal classes, respectively, and the different plant species as the abundance or presence data.

In addition, a quantitative network approach has been used to evaluate the structure of the interactions between fleshy-fruited plants, and mammalian and avian seed dispersers (Bascompte & Jordano, 2007). We pooled the number of interactions (i.e., the total number of samples in which at least a viable seed was found; if seeds of two species were found in the same sample it counted as two interactions; Vizentin-Bugoni et al., 2019), for each plant–frugivore pair (mammalian or avian) from all the animal-dispersed samples in the sampling transects and across the three sites throughout the season. The network structure was depicted as a bipartite graph and evaluated using basic parameters that represent complementary aspects of the structure of a mutualistic network (Peredo et al., 2013): connectance (C), the proportion of interactions detected relative to all the potential interactions in the network; linkage density to assess the average linkage level; nestedness (WNODF), the degree to which the interactions of the poorly connected species are a subset of the highly connected species; complementary specialization (H′_2_), a measure of niche complementarity between species; generality (G), a weighted average of the number of plants dispersed per frugivore; and vulnerability (V), a weighted average of the number of frugivores per plant. We calculated species–specific parameters to identify the number of partners of each plant and disperser species (degree), which species were the most selective (by means of d′) and which species were the most important of each trophic level (species strength). To test whether the parameters estimated from the empirical network differed significantly from networks with randomly interacting species, we compared the observed values with those of 1,000 random networks based upon a Patefield null model (Dormann, Fründ & Gruber, 2014). We also calculated network modularity (Q), which indicates the extent to which resistance or infectivity interactions can be partitioned into distinct groups, each of which has many internal interactions but few with other groups (Dormann & Strauss, 2014), identifying the main modules related to the roles of frugivorous guilds (mammals vs. birds). Moreover, null model comparison was calculated by means of *z*-scores, where *z*-scores values above two indicate a significant modularity.

All analyses were carried out using R software version 3.4.0 (R Development Core Team, 2016). Statistical tests and *Chao* estimators were performed with the vegan R package (Oksanen et al., 2007) and the graphs were obtained with ggplot2 (Wickham, 2010). Specifically, the network analyses and their graphs were performed using the bipartite R package (Dormann, Fründ & Gruber, 2014; Dormann & Strauss, 2014).

## Results

### Plant composition and seed disperser species

**Figure 2:**
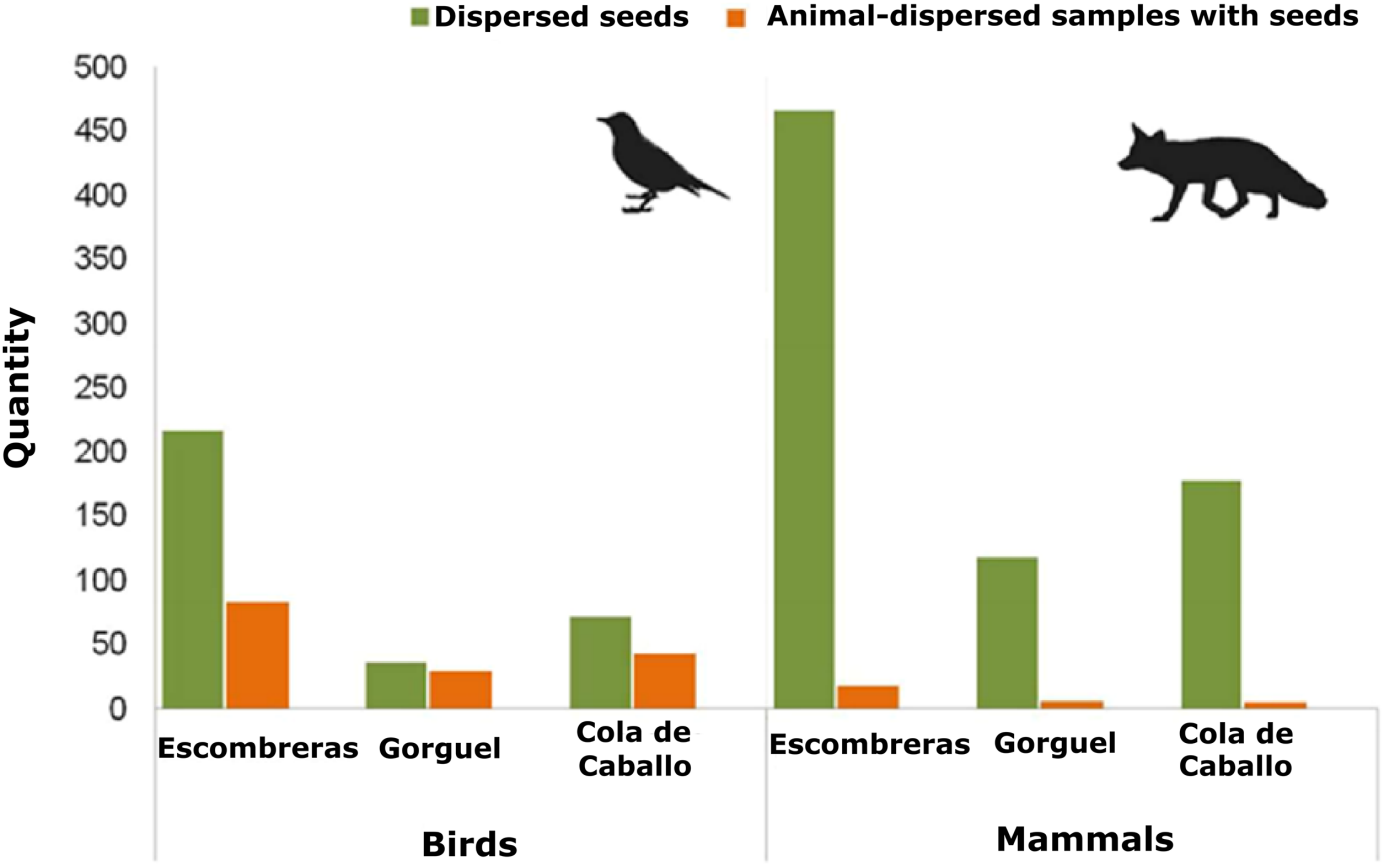
Quantity of animal-dispersed samples with seeds and seeds dispersed by mammals and birds in the three areas sampled within the Sierra de la Fausilla (SE Iberian Peninsula): dark green bars show the quantity of seeds; light orange bars show the quantity of animal-dispersed samples.

There were 12 fleshy-fruit species identified on the plots of vegetation studied here (Table S1). Mean density values of the fleshy-fruit species and of their dispersed seeds were similar among the three sampling sites (Density of fleshy-fruit species—Kruskal–Wallis χ^2^ = 0.104, d*f* = 2, *p* = 0.949; dispersed seed density—Kruskal–Wallis χ^2^ = 3.046, d*f* = 2, *p* = 0.218). Overall 650 animal-dispersed samples were collected from the SCI—Sierra de la Fausilla, of which 33.2% were mammalian and 63.1% were avian, with 3.7% of undetermined taxonomy. Escombreras was the sampling site with most seeds, followed by Cola de Caballo and Gorguel (Fig. 2; Table S2). Of the 216 mammalian faecal samples, only 29 contained seeds and these faeces were deposited by five species of mammals: beech marten *Martes foina* Erxleben.; badger *Meles meles* L.; fox *V. vulpes*; rabbit *Oryctolagus cuniculus* L. and hedgehog *Erinaceus europaeus* L. Significantly, most seeds were dispersed by carnivorous mammals like marten (43.44%) and fox (30.05%). Approximately, 38% of the samples collected from birds contained seeds (155 of 410 samples). Mammals dispersed a larger number of seeds than birds, 762 and 325 seeds, respectively, although this quantity was clearly biased by the consumption of figs (*Ficus carica* L.) by mammals. This human cultivated fruit tree usually has 100 seeds per dispersal unit and consequently, it is probable that the seeds observed in one faecal sample came from just one fig. In addition, we detected seed dispersal of Pygmy date palm (*Phoenix roebelenii* O’Brien), an exotic plant, by *V. vulpes* and *Martes foina* (Table S2).

Regarding birds, the disperser species was successfully identified in 92% of the seeds sampled (*n* = 299). We sequenced fragments amplified with different combinations of the four primers mentioned above (see “Data accessibility” for details about the sequenced strand), although only one strand was sequenced for most samples (COI-fsd-degF) as the length obtained was sufficiently long for species identification (Length range = 159–465 bp; mean = 233.34 ± 4.08 bp). DNA barcoding allowed us to identify eight birds dispersing seeds in our study area based on a similarity threshold >99%: *Erithacus rubecula* L.; *Phoenicurus ochruros* S. G. Gmelin; *Sylvia atricapilla* L.; *S. melanocephala* Gmelin; *S. undata* Boddaert.; *Turdus merula* L.; *T. philomelos* C. L. Brehm.; and *T. viscivorus* L.

**Figure 3:**
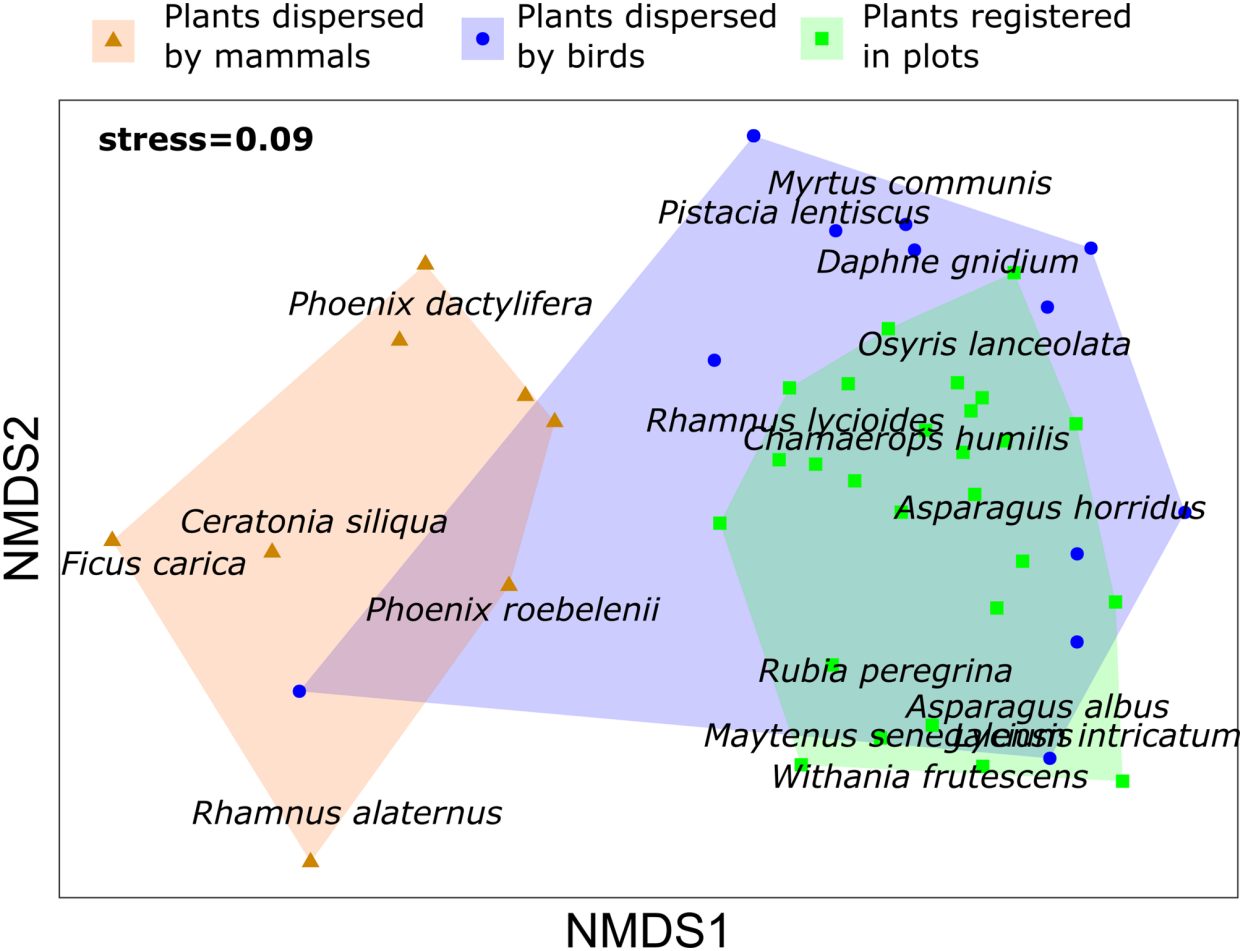
Non metric multidimensional scaling (NMDS) for plant species registered in vegetation samples and plant species dispersed by mammals and birds in Sierra de la Fausilla, Murcia Region.

Concerning the relationship between the composition of the plant community and the dispersed seeds, the density of fleshy-fruit shrubs did not show any relationship to the dispersed seed density (*R*^2^ = 0.073, *p* = 0.331), although we registered the highest values of fleshy-fruited shrubs density and dispersed seeds in Escombreras. The plant community registered in each plot of vegetation was dissimilar to the plant species dispersed by birds (PERMANOVA, *F* = 3.377, *R*^2^ = 0.082, adjusted *p*-value = 0.006) and by mammals (PERMANOVA, *F* = 10.142, *R*^2^ = 0.229, adjusted *p*-value = 0.003), and there were also significant differences in the plant species dispersed by each frugivorous guild (*F* = 5.355, *R*^2^ = 0.196, adjusted *p*-value = 0.003) (Table S3). However, NMDS showed a higher contribution of birds to disperse plant species occurring in the study sites (Fig. 3).

### Seed dispersal networks: identifying the key species in seed dispersal

**Figure 4:**
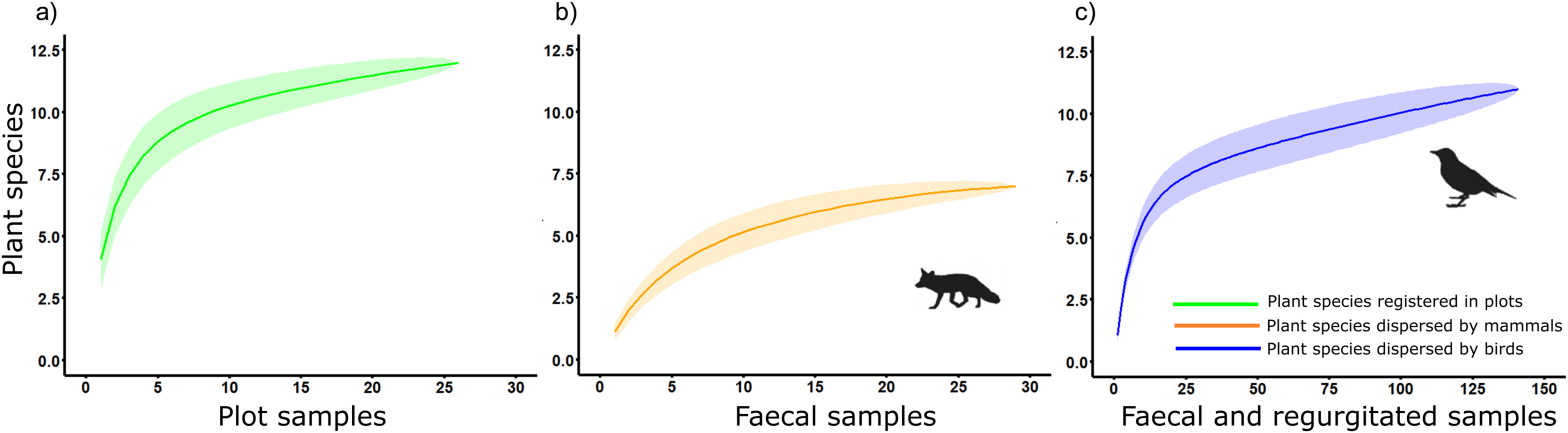
Accumulation curves on plant species detected considering its corresponding sampling effort in a Mediterranean ecosystem at Sierra de la Fausilla (SE Spain) for: (A) the sampling of native plants (green); (B) mammal faecal samples (orange); (C) faecal and regurgitated samples from birds (blue).

*Chao1* estimated true diversity as 12.25, 7, and 13 species for sampling of native plants, plant species dispersed by mammals and birds, respectively, while *Chao2* estimated the richness of the sample as 14, 7.25, and 15.5 for sampling of native plants, plant species dispersed by mammals and birds, respectively (Fig. 4). These results highlight the role of birds as main seed dispersers in the study area, at least in terms of the diversity of plant species dispersed.

**Figure 5:**
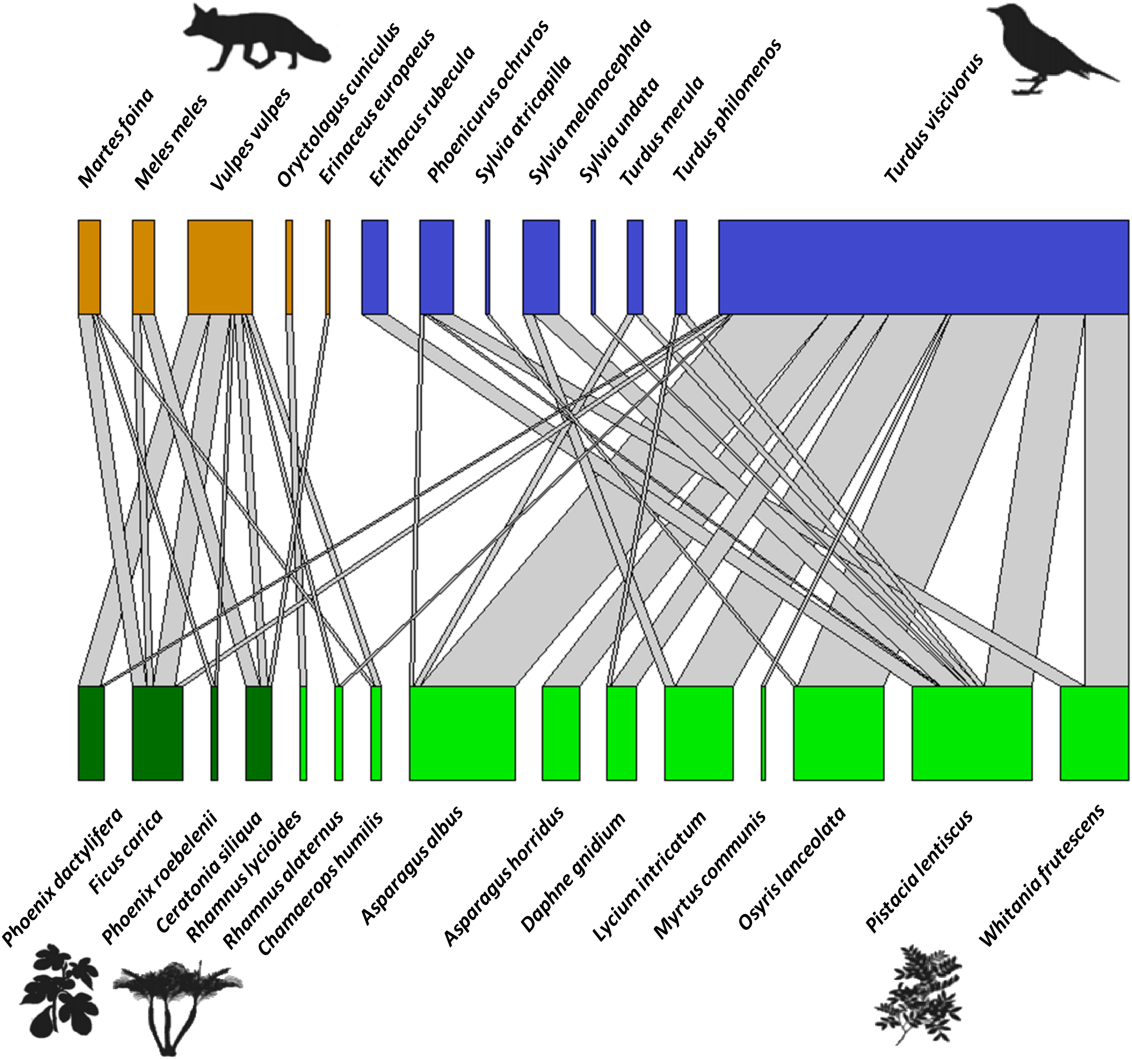
Bipartite network graph representing the proportion of dispersed seeds of fleshy-fruited species (bottom row), those dispersed by frugivore vertebrates (upper row) (based on 1,061 seeds, 181 interactions). Colors show the high complementary role of frugivore taxa (orange: mammals vs. blue: birds), and the different types of plants (light green: native plants vs. dark green: exotic and human-cultivated plants). Gray links show the proportion of interactions between each plant and each animal species, or in other words, the number of times that viable seeds of a plant species were found in animal-dispersed samples in a Mediterranean ecosystem at Sierra de la Fausilla (SE Spain).

The bipartite network between fleshy-fruit plants and vertebrates (mammals and birds) included a total of 181 interactions (with 1,061 dispersed seeds), showing strong biases in the distribution of these interactions (Fig. 5). From the perspective of the 15 plant species dispersed, *Pistacia lentiscus* was the most dominant species, with *Asparagus albus* L., *O. lanceolata*, *L. intricatum*, and *W. frutescens* among the most dispersed plants in a second order. *Pistacia lentiscus* was the species associated with the largest number of frugivores (*n* = 7). Regarding mammals, most of the dispersal events were done by *V. vulpes*, being also that dispersed the widest variety of seeds. In terms of the avian species, *T. viscivorus* was responsible for the 76.3% of the dispersal interactions by birds. Moreover, while *Pistacia lentiscus* was dispersed by a wide range of bird species, *L. intricatum* and *W. frutescens* were only dispersed by two species. From all the bird species identified, *T. viscivorus* was the dominant disperser and it interacted with the largest number of plant species. The species level parameters (Table S4) also showed that the most important fleshy-fruited plant of the priority habitat 5220* was *Pistacia lentiscus* and the most important seed disperser was *T. viscivorus* since they showed the highest values of degree and species strength. On the other hand, *Rhamnus lycioides* L. and *O. cuniculus* were the most selective species from the fleshy-fruited plants and seed dispersers, respectively, supported by the highest values of d’. This selectivity could be explained by the scarcity of *R. lycioides* seeds and the occasional frugivorous role of *O. cuniculus*.

Network parameters showed that there were a lower connectance (C) and linkage density in the seed dispersal network than expected under a null model (Table 1), since more than half of all the potential plant–frugivore links were not observed (Fig. 5). Nestedness (WNODF) was higher than expected in the network (Table 1), given that the interactions of poorly connected species (*S. undata*, *S. atricapilla* and *Erinaceus europaeus*) were highly nested within those of highly connected species (like *T. viscivorus* and *V. vulpes* Fig. 5). The degree of specialization within the network (H’_2_) was higher than the anticipated by the null model, probably because rarer plant species were almost exclusively dispersed by occasional frugivores, for example *O. cuniculus* only dispersed *R. lycioides* (represented in one module, Fig. S2). Modularity (Q) was 0.412, being significantly modular (*z*-scores = 15.942; Qnull = 0.161) and identifying five modules in the network (Fig. S2), where frugivorous taxa had a complementary role: whereas mammals dispersed especially exotic and human-cultivated plant species (represented in two modules), birds dispersed most representative plant species from habitat 5220*.

**Table 1:** Metrics of the seed dispersal network between plants and vertebrates (mammals and birds) in the coast of Cartagena (SE Spain), together with mean values and lower and upper confidence intervals from 1,000 networks calculated by a Patefield null model.

	Estimate	Null model			*t*	*p*
		Mean	Lower CI	Upper CI		
Connectance (C)	0.185	0.311	0.311	0.312	302.970	0.000
Nestedness (WNODF)	18.410	14.408	14.189	14.627	−35.847	0.000
Interaction strength asymmetry	−0.081	−0.094	−0.095	−0.093	−25.679	0.000
Specialisation asymmetry	0.215	0.043	0.039	0.047	−83.891	0.000
Linkage density	3.963	5.853	5.842	5.863	340.183	0.000
Specialization (H’2)	0.534	0.140	0.139	0.142	−534.091	0.000
Cluster coefficient HL*	0.536	0.735	0.733	0.737	200.013	0.000
Cluster coefficient LL**	0.246	0.454	0.452	0.455	282.434	0.000
Generality (G) HL	5.629	8.321	8.306	8.337	338.911	0.000
Vulnerability (V) LL	2.297	3.384	3.377	3.391	296.605	0.000

**Notes:**

Results of *t*-tests comparing observed and null-model values are also shown.

* HL, high level species (mammals and birds).

** LL, low level species (plants).

It is also relevant to note the high degree of complementarity among mammals and birds in the seed dispersal network, as *F. carica*, *Phoenix dactylifera* L. and *R. alaternus* L. were the only plants dispersed by members of both taxa (mammals and birds; Fig 5). This may be another factor to explain the high degree of specialization within the network. According to the generality (G), each frugivore dispersed an average of six plant species (Table 1). Vulnerability (V) was lower than generality (with an average of two dispersers per plant), since the average number of frugivores per plant was lower than the number of plants per frugivore, although both parameters were lower than in the null model (Table 1).

## Discussion

In this study we have evaluated the mammalian and avian agents responsible for seed dispersal in the SCI—Sierra de la Fausilla (Southeast of Spain). The ecological network obtained showed a strong functional complementarity between frugivores, as witnessed by the low levels of connectance and high levels of specialization. Moreover, birds seemed to be more important than mammals for seed dispersal of fleshy-fruited plants occurring in the study habitat. In conjunction, our results of seed dispersal by frugivores (mammals and birds) in the 5220* priority habitat allow us to conclude that both dispersal taxa fulfil a complementary role as suppliers of the seed dispersal service.

Birds have shown a complementary role to that of mammals, acting as the main dispersers of native vegetation in the shrubland habitat 5220*. Birds dispersed almost all plants occurring in the network (11 out of 15). Statistically significant differences found between plant species dispersed by birds and native vegetation in the study areas are probably due to plant species that were present in vegetation sampling but which did not occur in the seed dispersal network because they did not produce fruits during the study year (e.g., *Maytenus kursive*). Contrastingly, there were species which were not detected in vegetation sampling due to their low abundance in the study area, but their seeds were detected in the seed dispersal networks (e.g., *Myrtus communis* L.). This is corroborated by the *Chao* estimator values of plant species dispersed by birds higher than plant species detected in vegetation samplings. Furthermore, *Chamaerops humilis* L. was quite abundant in the study areas and it was not dispersed by birds since its seeds are only dispersed by mammals. We found more bird-dispersed samples than mammal samples (around 2/3 from birds). However, it would be expected to have a higher bias to bird-dispersed samples. This phenomenon could be caused due to the smaller size and more random location of bird faeces and regurgitations, as well as the fact that we sampled mammal faeces in dirt roads, which are known to be preferred by mammal for defecating (Suárez-Esteban, Delibes & Fedriani, 2013), and thus, these anthropogenic structures concentrate more mammal faeces than expected by random. The fact that *Pistacia lentiscus* was the most important plant species in the network is not rare (as showed by the highest species strength and because it formed one module in the network), as its lipid-rich fruits are an important part of the diet of frugivorous migratory birds (Verdú & García-Fayos, 2002) what is known to structure the functioning of communities in which inhabits (Herrera, 1984). The interaction strength in seed dispersal networks is related to frugivores functional traits (Pigot et al., 2016). Body mass is the trait of the avian dispersers with the highest quantitative impact on avian seed dispersal network (Pigot et al., 2016), while gape width conditions the size of fruit that bird species can consume (Wheelwright, 1985). These factors could explain why *T. viscivorus* was the main seed disperser, as it has the largest body mass and one of the greatest gape widths among all the bird species in the network (data on the functional traits of the dispersers can be found in Pigot et al., 2016). This result is corroborated by other studies in which *T. viscivorus* is shown to exert a relevant influence on seed dispersal of *Prunus mahaleb* L. seeds, responsible for 26.4% of the total seed dispersed (Jordano et al., 2007), on the dissemination of some species that inhabit temperate secondary forests in north-western Spain (Martínez, García & Obeso, 2008), and even on seed dispersal in semiarid oldfields to be restored (Martínez-López et al., 2019). Moreover, the barcoding technique used in this study was fundamental to detect the key role in seed dispersal of this species, which was not registered by bird surveys in the study area (F. Robledano & J.F. Calvo, 2016, personal communication) due to the imperfect detection of this avian species (Jiménez-Franco et al., 2019).

Our results are consistent with previous studies in which complementarity in mammalian and avian seed dispersal was also found (Escribano-Ávila et al., 2012; Jordano et al., 2007; Peredo et al., 2013). The plant species dispersed by mammals differed from the local flora in the SCI—Sierra de la Fausilla, as they were mainly involved in the dispersal of human-cultivated plants (*F. carica*, *Phoenix* spp. and *Ceratonia siliqua* L.). These results are consistent with the general patterns of frugivory (fruit consumption) described for carnivorous mammals. Carnivorous mammals show a preference for the consumption of large fruits with many seeds and high water content (Herrera, 1989). These fruit traits are not common in the Mediterranean shrublands of the 5220* habitat where plants usually produce small fruits with few seeds. However, these characteristics are common to fruits of species cultivated by humans, such as *F. carica*, and thus, they are frequently found in carnivorous mammal’s faeces (López-Bao & González-Varo, 2011; Matías et al., 2010), although it can be also found in bird faeces with a low frequency (Costa et al., 2014) as found in our study in the case of *T. viscivorus*. Furthermore, carnivorous mammals have wide home ranges (González-Varo et al., 2015 and references therein) which explains why we found *F. carica* and *C. siliqua* seeds, despite the fact that we did not detect such species in our vegetation samplings, since mammals are consuming these fruits in surroundings cultivated areas. Thus, carnivorous mammals transport seeds between different kinds of habitats (González-Varo et al., 2015 and references therein), contributing to the dissemination of exotic and agricultural plant species (Matías et al., 2010; Padrón et al., 2011). For instance, this is the case of the exotic *Phoenix roebelenii*, commonly used as ornamental plant and dispersed by *V. vulpes* and *Martes foina* in our study area. The invasion of exotic species is one of the main causes of the loss of diversity worldwide (Van Der Wal et al., 2008) and, for instance, *V. vulpes* has already been recognized as one of the principal seed dispersers of allochthonous plant species in Mediterranean areas (Guix et al., 2001). Therefore, the recognition of alien plants seed dispersal in an ecosystem of special priority interest is indicative of the need for further research to assess their potential impacts on those natural ecosystems and the implementation of control measures if necessary (Correia et al., 2017).

Management and restoration policies to preserve and restore 5220* habitat at a community level, should be mainly directed to enhance the role of birds in seed dispersal, given that they are the main dispersers of the native flora inhabiting this priority habitat. The role of mammals in seed dispersal of native flora of this habitat would be restricted to some particular species such as *C. humilis* (Fedriani & Delibes, 2011) and *Z. lotus* (Cancio et al., 2016). In terms of birds as restoration agents, they tend to deposit seeds under places where they perch, which may be branches of trees or even similar artificial structures (a “perch effect”: Pausas et al., 2006). Thus, birds concentrate the arrival of seeds in some environments (a “nucleation effect”: Verdú & García-Fayos, 1998). This behavior of frugivorous birds implies that they are not good dispersers in open environments, as it is the case of many degraded Mediterranean semiarid lands (see the review of Escribano-Ávila et al., 2015). Therefore, developing strategies to enhance the 5220* habitat restoration capability of birds in increasingly fragmented scenarios would be highly advisable. For instance, research into natural habitat restoration in degraded Mediterranean lands suggests installing artificial perches to promote the targeting of seeds to restoration sites (see review from Guidetti et al., 2016; Pausas et al., 2006). These measures should be executed in conjunction with the assessment of the plant regeneration process to test the role of frugivorous birds in plant establishment, as seed arrival does not guarantee successful vegetation recovery (Reid & Holl, 2013).

Spatio-temporal changes in fruit availability are common in ecosystems (García et al., 2013; Herrera, 1998). For instance, some Mediterranean fleshy-fruited plant species synchronize their fruit production over long periods (masting behavior: Herrera et al., 1998, 1994; Janzen, 1976). These temporal fluctuations in fruit availability could also mirror changes in the community of dispersers, with frugivorous species tracking the abundance of fruits (Tellería, Ramirez & Pérez-Tris, 2008). In this study, we sampled different sites within the 5220* priority habitat, but our field work was limited in time to only one year. Therefore, future studies should address seed dispersal over a longer temporal scale in order to fully fathom out the role of such biotic interactions in the maintenance of shrubland communities in this Mediterranean semiarid ecosystem. We have measured seed dispersal by mammals and birds in the 5220* habitat; however, this does not guarantee the contribution of all registered animal species to the maintenance of vegetation of this habitat. A given seed dispersers is only considered effective if its activity results in new adults of the dispersed plant species (seed dispersal effectiveness; Schupp, 1993). Thus, next investigations should measure how effective are the different seed dispersers, for instance by performing germination tests and/or seedling survival rates. Despite the fact that morphological identification of mammal faeces has been extensively used when study seed dispersal (see González-Varo et al., 2015 and references therein), some investigations have detected misidentification cases between some species (Dalén, Götherström & Angerbjörn, 2004; Davison et al., 2002). Thereby, the proposed approach could be improved by using molecular techniques to identify faeces of mammals, or at least, a subset of the samples in order to estimate the misidentification risk.

## Conclusions

These results represent the first assessment of a quantitative seed dispersal network in the priority habitat 5220* at a community level. By combining ecological and genetic tools, we have been able to discern different roles of mammals and birds in seed dispersal of native fleshy-fruited shrubs that inhabit an emblematic protected area in the Southeast of the Iberian Peninsula. Overall, our inferences highlight the functional complementarity between seed dispersal taxa, with birds dispersing key species of this habitat, while mammals mainly disperse human-cultivated species, including exotic species. These results provide information that is relevant to devise management policies for this highly threatened habitat, particularly as plant-animal interactions help to prevent the collapse of plant communities when they are faced with habitat degradation (Montoya et al., 2008).

## Supplemental Information

10.7717/peerj.7609/supp-1 Supplemental Information 1 Matrix for PERMANOVA and NMDS. Plant species dispersed by mammals and birds (in each area and sampling date), and plant community detected in the vegetation samplings

10.7717/peerj.7609/supp-2 Supplemental Information 2 DNA sequences from bird seed dispersers identified in the study.

10.7717/peerj.7609/supp-3 Supplemental Information 3 Matrix of seed dispersal events between animals (birds and mammals) and fleshy-fruited plants.

10.7717/peerj.7609/supp-4 Supplemental Information 4 Number of seeds dispersed by birds and mammals per dispersal event.

10.7717/peerj.7609/supp-5 Supplemental Information 5 Vegetation sampling. Plant species detected in each surveyed plots in the three study areas

10.7717/peerj.7609/supp-6 Supplemental Information 6 Supplemental Information

## Additional Information and Declarations

### DNA Deposition

The following information was supplied regarding the deposition of DNA sequences: The DNA sequences are available in the Supplemental File.

### Data Availability

The following information was supplied regarding data availability: The raw data are available in the Supplemental Files.
