# Supplementary material for: An integrative approach to discern the seed dispersal role of frugivorous guilds in a Mediterranean semiarid priority habitat
Source: PeerJ. 2019 Oct 11;7:e7609. doi: 10.7717/peerj.7609 (PMC6792472; doi:10.7717/peerj.7609)
Supplement: Supplemental Information 6 [file peerj-07-7609-s006.docx]

***Supplementary Information for***

**An integrative approach to discern the seed dispersal role of frugivorous guilds in a Mediterranean semiarid priority habitat**

**Authors:** Acosta-Rojas, D.C.^1, 2^, Jiménez-Franco, M.V.^3^, Zapata, V.M.^4^, & De la Rúa, P.^1^, Martínez-López, V.^1, 4†^

*^1^ Department of Zoology and Physical Anthropology. Faculty of Veterinary. University of Murcia. Murcia, 30100 Spain; ^2^Senckenberg Biodiversity and Climate Research Centre (BiK-F). Frankfurt am Main, Germany; ^3^Department of Applied Biology, Faculty of Experimental Sciences, Miguel Hernández University, 03202 Elche, Spain; ^4^Department of Ecology and Hydrology. Faculty of Biology, University of Murcia. Murcia, 30100 Spain.*

**Corresponding author email**: [vicente.martinez2@um.es](mailto:dianacarolina.acostar@um.es)

**Fig. S1** Estimated distribution map of 5220 Habitat* in Spain. (Source: Atlas de los Hábitat de España, 2005).

**Fig. S2** Interaction matrix of dispersed seeds of fleshy-fruited species featuring modules.

**Table S1.** Density of fleshy-fruited shrub species (individuals/100m^2^) recorded in vegetation sampling of habitat 5220* of Sierra de la Fausilla (Murcia Region - Spain).

**Table S2.** Plant species dispersed by mammals and birds in Sierra de la Fausilla (Murcia Region, Spain). Values are the amount of seeds of each plant species found in animal-dispersed samples (i.e. faeces of mammals and faeces and regurgitations of birds).

**Table S3.** Permutational Multivariate Analysis of Variance PERMANOVA with Bonferroni correction, using Bray Curtis distances and 9999 permutation of plant communities dispersed by mamals, birds and censed in plots.

**Table S4.** Species-specific level parameters of the network between fleshy-fruited plants and vertebrates (mammals and birds) in the coast of Cartagena (SE Spain). The highest values for each parameter are shown in bold.

**Fig. S1** Estimated distribution map of 5220 Habitat* in Spain. (**Source:** Atlas de los Hábitat de España, 2005).

**
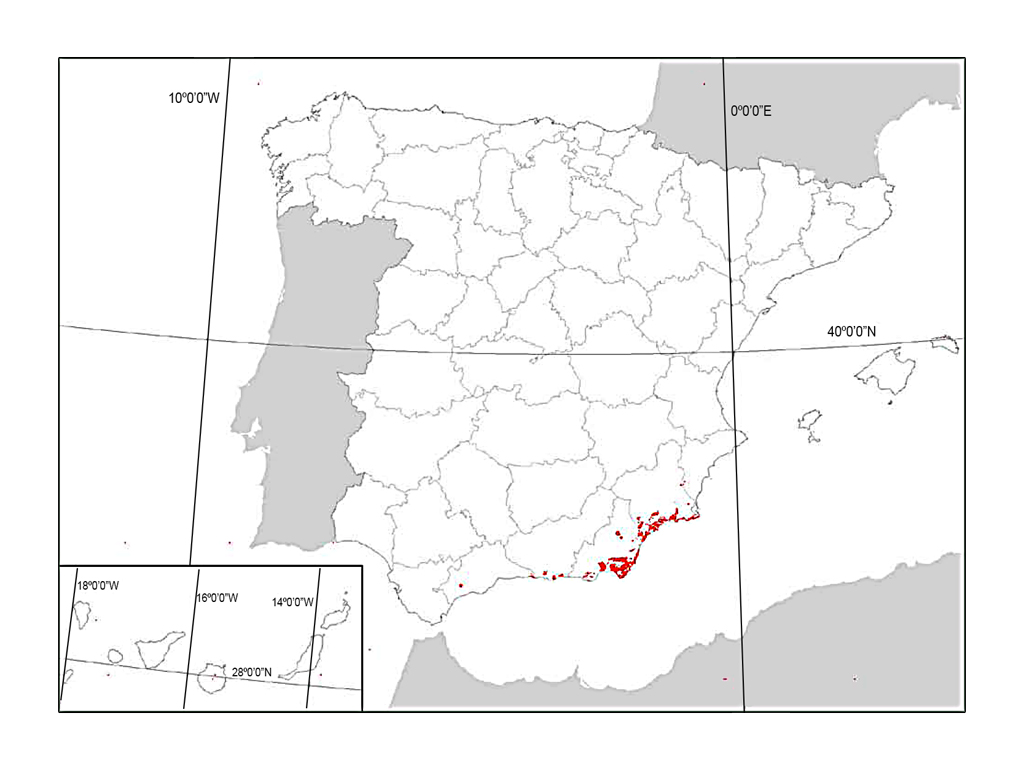
**

**Fig. S2** Interaction matrix of dispersed seeds of fleshy-fruited species featuring modules (red boxes delineate five modules). Darker blue squares indicate more observed interactions.

**
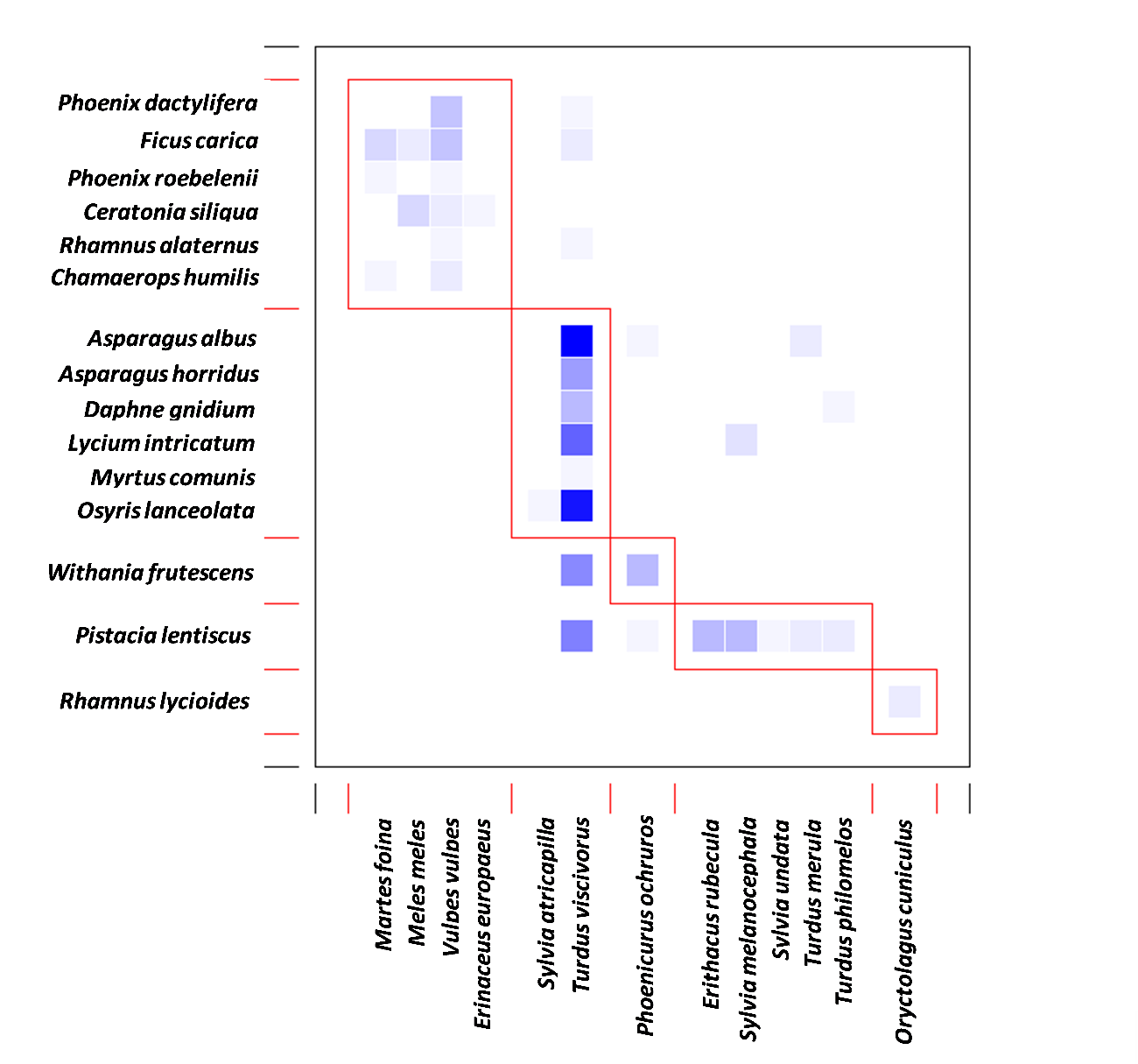
**

**Table S1.** Density of fleshy-fruited shrub species (individuals/100m^2^) recorded in vegetation sampling of habitat 5220* of Sierra de la Fausilla (Murcia Region - Spain).

| \| **Plants** \| \| **Sampling site** \| \| \| \| --- \| --- \| --- \| --- \| --- \| \| **Family** \| **Species** \| **Escombreras** \| **Gorguel** \| **Cola de Caballo** \| \| Anacardiaceae \|  \|  \|  \|  \| \|  \| *Pistacia lentiscus* \| - \| 12 \| 17 \| \| Arecaceae \|  \|  \|  \|  \| \|  \| *Chamaerops humilis* \| 1 \| 20 \| 31 \| \| Asparagaceae \|  \|  \|  \|  \| \|  \| *Asparagus albus* \| 64 \| 1 \| 1 \| \|  \| *Asparagus horridus* \| 17 \| 28 \| 7 \| \| Celastraceae \|  \|  \|  \|  \| \|  \| *Maytenus senegalensis* \| 9 \| - \| 5 \| \| Rhamnaceae \|  \|  \|  \|  \| \|  \| *Rhamnus alaternus* \| - \| - \| 2 \| \|  \| *Rhamnus lycioides* \| 1 \| 2 \| 17 \| \| Rubiaceae \|  \|  \|  \|  \| \|  \| *Rubia peregrina* \| 2 \| - \| - \| \| Santalaceae \|  \|  \|  \|  \| \|  \| *Osyris lanceolata* \| 2 \| 41 \| 13 \| \| Solanaceae \|  \|  \|  \|  \| \|  \| *Lycium intricatum* \| 18 \| - \| - \| \|  \| *Withania frutescens* \| 8 \| - \| 2 \| \| Thymelaeaceae \|  \|  \|  \|  \| \|  \| *Daphne gnidium* \| - \| - \| 1 \| \| **Density (average ± standard deviation)** \| \| 10 ± 18. 1 \| 8.6 ± 13.8 \| 8 ± 9.6 \|   **Table S2.** Plant species dispersed by mammals and birds in Sierra de la Fausilla (Murcia Region, Spain). Values are the amount of seeds of each plant species found in animal-dispersed samples (i.e. faeces of mammals and faeces and regurgitations of birds).   \|  \| \| \| \| \| --- \| --- \| --- \| --- \| \| **Plants** \| \| **Seed dispersers** \| \| \| **Family** \| **Species** \| **Mammals** \| **Birds** \| \| Anacardiaceae \|  \|  \|  \| \|  \| *Pistacia lentiscus* \| - \| 45 \| \| Arecaceae \|  \|  \|  \| \|  \| *Chamaerops humilis* \| 7 \| - \| \|  \| *Phoenix roebelenii* \| 3 \| - \| \|  \| *Phoenix dactylifera* \| 10 \| 1 \| \| Asparagaceae \|  \|  \|  \| \|  \| *Asparagus albus* \| - \| 45 \| \|  \| *Asparagus horridus* \| - \| 15 \| \| Fabaceae \|  \|  \|  \| \|  \| *Ceratonia siliqua* \| 14 \| - \| \| Moraceae \|  \|  \|  \| \|  \| *Ficus carica* \| 723 \| 10 \| \| Myrtaceae \|  \|  \|  \| \|  \| *Myrtus communis* \| - \| 3 \| \| Rhamnaceae \|  \|  \|  \| \|  \| *Rhamnus alaternus* \| 1 \| 1 \| \|  \| *Rhamnus lycioides* \| 4 \| - \| \| Santalaceae \|  \|  \|  \| \|  \| *Osyris lanceolata* \| - \| 36 \| \| Solanaceae \|  \|  \|  \| \|  \| *Lycium intricatum* \| - \| 112 \| \|  \| *Withania frutescens* \| - \| 49 \| \| Thymelaeaceae \|  \|  \|  \| \|  \| *Daphne gnidium* \| - \| 8 \| |
| --- | --- | --- | --- | --- | --- | --- | --- | --- | --- | --- | --- | --- | --- | --- | --- | --- | --- | --- | --- | --- | --- | --- | --- | --- | --- | --- | --- | --- | --- | --- | --- | --- | --- | --- | --- | --- | --- | --- | --- | --- | --- | --- | --- | --- | --- | --- | --- | --- | --- | --- | --- | --- | --- | --- | --- | --- | --- | --- | --- | --- | --- | --- | --- | --- | --- | --- | --- | --- | --- | --- | --- | --- | --- | --- | --- | --- | --- | --- | --- | --- | --- | --- | --- | --- | --- | --- | --- | --- | --- | --- | --- | --- | --- | --- | --- | --- | --- | --- | --- | --- | --- | --- | --- | --- | --- | --- | --- | --- | --- | --- | --- | --- | --- | --- | --- | --- | --- | --- | --- | --- | --- | --- | --- | --- | --- | --- | --- | --- | --- | --- | --- | --- | --- | --- | --- | --- | --- | --- | --- | --- | --- | --- | --- | --- | --- | --- | --- | --- | --- | --- | --- | --- | --- | --- | --- | --- | --- | --- | --- | --- | --- | --- | --- | --- | --- | --- | --- | --- | --- | --- | --- | --- | --- | --- | --- | --- | --- | --- | --- | --- | --- | --- | --- | --- | --- | --- | --- | --- | --- | --- | --- | --- | --- | --- | --- | --- | --- | --- | --- | --- | --- | --- | --- | --- | --- | --- | --- | --- | --- | --- | --- | --- | --- | --- | --- | --- | --- | --- | --- | --- | --- | --- | --- | --- | --- | --- | --- | --- | --- | --- | --- | --- |

**Table S3.** Permutational Multivariate Analysis of Variance PERMANOVA with Bonferroni correction, using Bray Curtis distances and 9999 permutation of plant communities dispersed by mammals, birds and censed in plots.

|  | **Degree freedom** | **Sum of squares** | **Average sum of squares** | **F** | **R^2^** | **p-value** | **p-value adjusted** |
| --- | --- | --- | --- | --- | --- | --- | --- |
| *General* | 2 | 3.822 | 1.911 | 6.179 | 0.208 | <0.001 |  |
| Residuals | 47 | 14.535 | 0.309 |  | 0.792 |  |  |
| Total | 49 | 18.357 |  |  | 1 |  |  |
| --- |  |  |  |  |  |  |  |
| *Pair comparison* |  |  |  |  |  |  |  |
| Plants dispersed by mammals *vs.*  Plants dispersed by birds |  |  |  | 5.355 | 0.196 | 0.001 | 0.003 |
| Plants dispersed by mammals *vs.*  Plants of semiarid shrubs |  |  |  | 10.142 | 0.229 | 0.001 | 0.003 |
| Plants dispersed by birds *vs.*  Plants of semiarid shrubs |  |  |  | 3.377 | 0.082 | 0.002 | 0.006 |

**Table S4.** Species-specific level parameters of the network between fleshy-fruited plants and vertebrates (mammals and birds) in the coast of Cartagena (SE Spain). The highest values for each parameter are shown in bold

| **Plants** | **Degree** | **Species strength** | **d’** |
| --- | --- | --- | --- |
| *Phoenix dactylifera* | 2 | 0.342 | 0.455 |
| *Ficus carica* | 4 | 1.351 | 0.458 |
| *Phoenix roebelenii* | 2 | 0.222 | 0.420 |
| *Ceratonia siliqua* | 3 | 1.778 | 0.711 |
| *Rhamnus lycioides* | 1 | 1.000 | **1.000** |
| *Rhamnus alaternus* | 2 | 0.064 | 0.056 |
| *Chamaerops humilis* | 2 | 0.278 | 0.436 |
| *Asparagus albus* | 3 | 0.841 | 0.194 |
| *Asparagus horridus* | 1 | 0.088 | 0.100 |
| *Daphne gnidium* | 2 | 0.395 | 0.105 |
| *Lycium intricatum* | 2 | 0.442 | 0.158 |
| *Myrtus comunis* | 1 | 0.009 | 0.000 |
| *Osyris lanceolata* | 2 | 1.212 | 0.228 |
| *Pistacia lentiscus* | **7** | **4.093** | 0.363 |
| *Withania frutescens* | 2 | 0.884 | 0.308 |
| **Dispersers** |  |  |  |
| *Martes foina* | 3 | 1.119 | 0.634 |
| *Meles meles* | 2 | 0.714 | 0.671 |
| *Vulpes vulpes* | 6 | 3.238 | 0.734 |
| *Oryctolagus cuniculus* | 1 | 1.000 | **1.000** |
| *Erinaceus europaeus* | 1 | 0.143 | 0.443 |
| *Erithacus rubecula* | 1 | 0.212 | 0.481 |
| *Phoenicurus ochruros* | 3 | 0.433 | 0.448 |
| *Sylvia atricapilla* | 1 | 0.040 | 0.079 |
| *Sylvia melanocephala* | 2 | 0.370 | 0.395 |
| *Sylvia undata* | 1 | 0.030 | 0.000 |
| *Turdus merula* | 2 | 0.130 | 0.159 |
| *Turdus philomelos* | 2 | 0.186 | 0.238 |
| *Turdus viscivorus* | **11** | **7.385** | 0.403 |

**References**

Ministerio de Agricultura y Pesca, Alimentación y Medio Ambiente (MAPAMA). (2005). Atlas de los Hábitat de España. Spain. <http://www.mapama.gob.es>
